# Probiotics to improve the gut microbiome in premature infants: are we there yet?

**DOI:** 10.1080/19490976.2023.2201160

**Published:** 2023-04-30

**Authors:** Emily M. Mercer, Marie-Claire Arrieta

**Affiliations:** a Department of Physiology and Pharmacology, University of Calgary, Calgary, Alberta, Canada; b Department of Pediatrics, University of Calgary, Calgary, Alberta, Canada; c International Microbiome Center, University of Calgary, Calgary, Alberta, Canada

**Keywords:** Gut microbiome, probiotic, prematurity, preterm infants, microbial succession, microbiome maturation, early life, neonatal intensive care

## Abstract

Gut microbiome maturation in infants born prematurely is uniquely influenced by the physiological, clinical, and environmental factors surrounding preterm birth and early life, leading to altered patterns of microbial succession relative to term infants during the first months of life. These differences in microbiome composition are implicated in acute clinical conditions that disproportionately affect preterm infants, including necrotizing enterocolitis (NEC) and late-onset sepsis (LOS). Probiotic supplementation initiated early in life is an effective prophylactic measure for preventing NEC, LOS, and other clinical concerns relevant to preterm infants. In parallel, reported benefits of probiotics on the preterm gut microbiome, metabolome, and immune function are beginning to emerge. This review summarizes the current literature on the influence of probiotics on the gut microbiome of preterm infants, outlines potential mechanisms by which these effects are exerted, and highlights important clinical considerations for determining the best practices for probiotic use in premature infants.

## Introduction

Colonization and maturation of the infant gut microbiome typically occur in a conserved, stepwise fashion.^1–3^ Deviations from these successional patterns have been implicated in altered developmental programming of several key physiological systems, heightening the risk of the emergence of health impairments in childhood and adolescence.^4^ In infants born prematurely (<37 weeks gestational age (GA)), the first weeks to months of life are characterized by a unique set of microbial colonization patterns, prior to merging with those of term-born infants around 40 weeks postmenstrual age (PMA) or term-equivalent age.^5–8^ During this time, preterm infants endure frequent clinical interventions that can collaterally modify the gut microbiome (e.g., antibiotics, delayed enteral feeds, and stress-inducing procedures). These microbiome alterations may subsequently contribute to the increased vulnerability of preterm infants to developing necrotizing enterocolitis (NEC)^9–13^ and late-onset sepsis (LOS)^14–17^ compared to infants born at term. Although this period of delayed and altered microbial colonization is relatively short in duration, the consequences may be far-reaching, influencing several homeostatic signaling mechanisms during critical developmental stages that occur in early life.^4,18^

Probiotics, defined as live organisms that provide health benefits to the host when consumed in adequate quantities,^19^ are often used in the premature infant population as a prophylactic measure against NEC, LOS, feeding intolerance, and all-cause mortality.^20–26^ This practice remains a matter of ongoing debate due to concerns regarding the safety and efficacy of using live microbes as therapeutics for infants born prematurely, who are immunocompromised.^27–33^ Notwithstanding, probiotic administration has increased dramatically in neonatal intensive care units (NICUs) around the world as part of standard clinical care. Initial efforts have started to characterize the effects of probiotic treatment on the preterm infant gut microbiome.^34–41^ Recent work by our group^37,42^ and others^34,36^ has shown that probiotic strains, specifically those belonging to the bacterial genus, *Bifidobacterium*, significantly shift the composition of the preterm microbiome, accelerating microbiome maturation to a state more comparable to term-born infants, and facilitating health-promoting changes in immune and metabolic parameters. Furthermore, some probiotic strains have been shown to successfully engraft into the preterm microbiome,^34–37,39–41^ raising the possibility that these changes may be relevant beyond the prevention of acute clinical conditions, conferring long-lasting benefits to both the gut microbiome and host physiology even after treatment cessation.

In this review, we provide an overview of the maturational patterns of the preterm infant gut microbiome and how these differ from term infants, evaluate the ecological effects of probiotic supplementation on the preterm microbiome and host physiology, outline the mechanisms by which probiotics may induce health-promoting shifts in this population, and discuss clinical considerations for employing probiotics as a therapeutic strategy to ameliorate microbiome alterations in early life. Subsequently, we provide recommendations for future research required to better inform the development and implementation of probiotic supplementation as part of standard clinical care in NICUs.

## Gut microbiome maturation patterns in preterm infants

Postnatal colonization of the infant gastrointestinal tract with microbes represents a primary successional event, in which a previously uncolonized environment undergoes a dynamic, sequential process of ecosystem establishment. The earliest colonizers, referred to as pioneer species, modify the environment of the infant gut and exert influences on the order, timing, and success of subsequent colonizers in an ecological process known as priority effects.^43,44^ With the successive arrival of additional species, ecosystem modifications continue to occur, resulting in the occupation of ecological niches. This process is characterized by the formation of metabolic cross-feeding networks and competitive exclusion of new species, including pathogens, which typically lack the ability to successfully integrate into a resilient and stable gut microbial community.^45^ In parallel, environmental factors unique to different regions of the gastrointestinal tract, such as temperature, motility, pH, oxygenation, and nutrient availability, represent additional layers of selective pressure exerted on prospective colonizers.^46^ Ultimately, these dynamic ecological processes lead to the establishment of a taxonomically and functionally diverse climax community comprised primarily of strict anaerobes at approximately 3 years of age, which remains stable into adulthood.^47^

**Figure 1.**
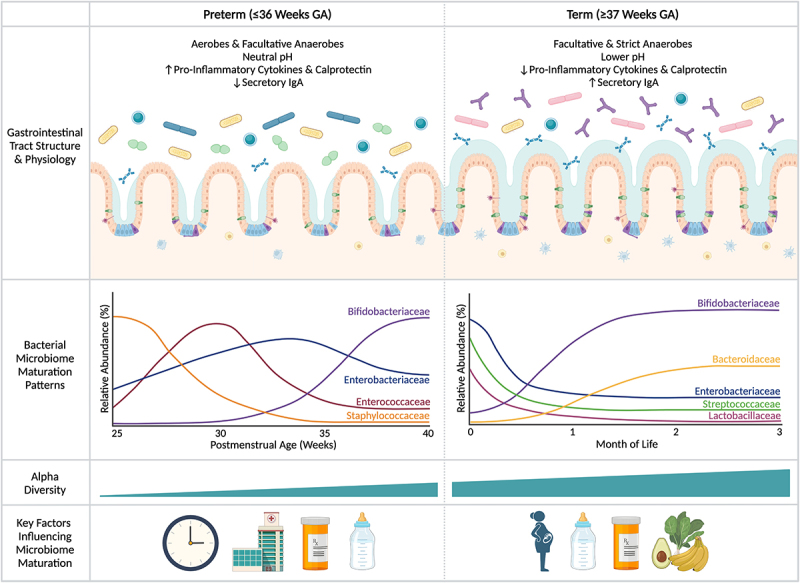
Differences in gastrointestinal physiology, bacterial microbiome maturation patterns, and key early life factors contributing to establishment of the gut microbiome in infants born prematurely versus at term. The gut microbiome of infants born prematurely displays unique maturation patterns in the first weeks to months of life relative to term-born infants due to differences in the maturity of the gastrointestinal tract and early-life exposures. With prematurity, the gastrointestinal tract is markedly underdeveloped at birth, resulting in broad structural and functional delays that may contribute to microbial colonization patterns. This includes immaturity of the intestinal epithelium, under-expression of tight junction proteins, and a lack of mature goblet cells leading to a patchy mucus layer, among other factors – all of which influence intestinal barrier function and reduce resistance to pathogen colonization. Simultaneously, the immune system of premature infants displays immaturities, including fewer mature immune cells surveying the gut, reduced secretory IgA expression, and higher levels of pro-inflammatory cytokines and the inflammatory marker, calprotectin, both giving rise to and reflecting potentially maladaptive responses to microbial antigens that typically guide the development of homeostatic immune responses in term infants. Together with the influence of factors such as gestational age at birth, prolonged hospitalization, increased antibiotic exposure, and delayed enteral feeds, the gut microbiome of preterm infants is colonized by a higher proportion of aerobic and facultative anaerobic bacteria in the first weeks of life and displays lower alpha-diversity. This leads to aberrant successional patterns characterized by Staphylococcaceae, Enterococcaceae, and Enterobacteriaceae dominance prior to achieving communities abundant in Bifidobacteriaceae around 40 weeks postmenstrual age (PMA) or term-equivalent age. The delayed membership of Bifidobacteriaceae also contributes to the higher intestinal pH observed in preterm infants, as the metabolic activities of Bifidobacteriaceae lead to the production of metabolites that effectively lower intestinal pH, such as acetic acid. Subsequently, microbiome maturation patterns largely follow those of term infants in the first months of life. GA, gestational age; IgA, immunoglobulin A.

Unlike term infants, who are initially colonized by microbes originating either from vaginal secretions and maternal feces when vaginally delivered or from the skin if born by Cesarean (C)-section,^48,49^ preterm infants are primarily colonized by hospital-associated microbes.^50,51^ This includes the predominance of potential pathobionts, such as *Staphylococcus* spp., *Enterococcus* spp., *Escherichia* spp., and *Klebsiella* spp.^5–7,52^ These divergent early colonization patterns arise in large part due to peri- and post-natal differences that occur more frequently in infants born prematurely, including elevated C-section rates, increased antibiotic use, delayed enteral feeds, and prolonged hospital stays.^53^ Multiple longitudinal studies on premature infants have consistently shown that the maturational patterns of the preterm gut microbiome are strongly associated with PMA.^5,54^ Early gut microbial communities are dominated by Staphylococcaceae (<30 weeks PMA), then transition to communities abundant in Enterococcaceae (30 weeks PMA) and Enterobacteriaceae (35 weeks PMA), prior to achieving Bifidobacteriaceae or Clostridiaceae dominance around 40 weeks PMA, depending on whether the infant is fed human or formula milk, respectively.^5,6^ In parallel, alpha-diversity increases over time, but is generally lower than in infants born at term (Figure 1).^52^ Subsequently, preterm infants converge into microbial maturation patterns characteristic of term infants, displaying less distinguishable differences in microbiome composition with age.^5^

In contrast to the bacterial microbiome, the fungal microbiome (mycobiome) of preterm infants exhibits stochastic changes over time, primarily being comprised of *Candida* spp. in the first weeks to months of life, before transitioning to *Saccharomyces* spp. dominance with the introduction of solid foods.^7,36,37,55,56^ This is comparable to patterns observed in term infants, although term infants often harbor other fungal colonizers in addition to *Candida* spp. early in life, such as *Debaromyces* spp., *Cryptococcus* spp., and *Malassezia* spp.^57–61^ Fungi have been identified as integral members contributing to microbial community assembly dynamics in the preterm microbiome.^7^ A sophisticated ecological analysis in preterm infants showed that *Candida* spp. inhibited both *Klebsiella* spp. and *Escherichia* spp. (members of Enterobacteriaceae), while the pioneer colonizer, *Staphylococcus* spp., imparted inhibitory effects on *Candida* spp.^7^ In contrast, our group did not observe any influence of *Candida* spp. on bacterial microbiome assembly in a probiotic intervention study in extremely preterm infants, but this may be due to the strong anti-*Candida* effect demonstrated in infants receiving probiotics.^37^ Together, this suggests that fungal colonizers are dynamically involved in gut microbiome assembly in preterm infants, albeit displaying less pronounced compositional differences in early life relative to term infants, and that these influences may be modified by probiotic supplementation.

Beyond bacteria and fungi, little is known about the presence and role of other microbiome members in infants born prematurely. For example, Archaea were observed in low abundance predominantly during the second month of life in one longitudinal study,^7^ but were undetectable in another.^36^ Comparably, studies examining the viral microbiome (virome) in preterm infants are sparse,^36,62^ but stochastic changes similar to those observed in the mycobiome and high interindividual variation have been described.^62^ Interestingly, recent research identified an association between the virome and NEC in premature infants; specifically, reductions in beta-diversity and the emergence of viral signatures characterized by over 100 viral contigs preceding the onset of NEC. These contigs were differentially associated with specific bacterial taxa; for example, positive associations were found with *Escherichia* spp. and *Streptococcus* spp. and negative associations with *Bifidobacterium* spp.^62^ This is particularly relevant considering probiotics containing *Bifidobacterium* have been shown to reduce the incidence of NEC,^20–23,25^ suggesting that the gut virome also influences the clinical course of preterm infants in a manner that could be modified by probiotic supplementation. These findings reiterate the importance of considering inter-kingdom interactions in gut microbiome assembly dynamics.

The preterm gut microbiome is also strongly influenced by the anatomical and functional differences in the gastrointestinal tract due to the developmental immaturities displayed across organ systems with preterm birth.^63^ The premature gastrointestinal tract displays shorter crypts and villi leading to limited digestive capacity;^64^ reduced production of mucus and antimicrobial peptides due to the presence of fewer or immature goblet^65^ and Paneth cells,^66^ respectively; increased intestinal permeability due to reduced expression of tight junction proteins;^67^ elevated oxygen levels as a result of delayed enteral feeds and reduced colonization with strict anaerobes;^68^ and decreased motility and gut hormone secretion, in part due to prolonged parenteral nutrition (Figure 1).^69,70^ In parallel, preterm infants are considered immunocompromised,^32,33^ placing them at a greater risk of developing serious infections or gastrointestinal conditions, such as LOS and NEC.^71^ Beyond these acute conditions, the combination of gastrointestinal and immune immaturity observed in the preterm population may lead to aberrant immune development through increased translocation of pathogenic microbes, microbial by-products, and other constituents from the intestines to the internal milieu.^72^ This complex scenario renders preterm infants vulnerable to harboring an aberrant microbiome and experiencing divergent developmental processes relative to term-born infants. It remains largely unknown if the profound differences in the preterm gut microbiome contribute to the unique acute and chronic conditions more commonly affecting infants and children born prematurely. However, probiotic intervention studies provide a unique lens to investigate how microbiome modulation through probiotic use may influence the clinical course and long-term health outcomes in this unique pediatric population.

## The influence of probiotic supplementation on the preterm gut microbiome and host physiology

The overall findings of clinical trials examining probiotic supplementation for the prevention of NEC, LOS, feeding intolerance, and all-cause mortality in preterm infants have resulted in considerable support for probiotic use in the past decade. While evidence in favor of probiotic supplementation is variable depending on the timing of initiation, duration, probiotic strain(s) used, and health outcome(s) assessed, clinical uptake of this practice continues to increase in NICUs around the world.^20–25^ Most arguments against probiotic supplementation center around the vulnerability of preterm infants to infection and uncertainty regarding optimal probiotic formulations and doses.^27–31,73^ Under this lens, an in-depth analysis of the consequences of probiotic use on the preterm gut microbiome can provide important information to help reconcile this debate. Without understanding the larger ecological processes that occur when supplementing a developing ecosystem with prospective colonizers, true risk assessment and optimization of probiotic formulations for use in the preterm population cannot be achieved. Here, we provide a summary of both randomized controlled trials (RCTs) and observational studies examining the microbiome alterations that occur with probiotic use in preterm infants. Given the large phylogenetic and metabolic differences between genera commonly used in probiotic preparations for preterm infants and the distinct effects that each taxon exerts on the preterm gut microbiome, we have divided this section taxonomically into the following subsections: *Bifidobacterium* spp., Lactobacillaceae, multi-genera bacterial formulations, and *Saccharomyces* spp. (Table 1). Strain designations are included when defined in the original study and trade names are used when several studies examined the same probiotic product.

**Table 1.** Gut microbiome and functional alterations observed with probiotic supplementation in infants born prematurely.

Author, Location and Study Type	Study Design^a^	Probiotic Strains^b^ and Treatment Groups	α- and β-Diversity	Abundance	Functional Changes^c^
***Bifidobacterium*** spp.					
Athalye-Jape *et al*., 2022^74^ Australia RCT (DB)	GA: <28 weeks Frequency: Daily until 37 weeks PMA Dose: 3 × 10^9^	P1: *B. breve M*-16 V (n = 87) P2: *B. breve M*-16 V, *B. longum* subsp. i*nfantis M*-63, and B. *longum* subsp. *longum* BB536 (n = 86) C: From Patole *et al*., 2014 (*n* = 29)	α: ns β: Δ	↑ *Bifidobacterium* and ↓ *Streptococcus* and *Clostridium sensu stricto 1* in PBX ↑ *B. breve* and *B. bifidum* in P1 ↑ *B. longum*, *B. reuteri*, *B. pseudocatenulatum*, *Streptococcus pyogenes* and *Gardnerella vaginalis* in P2	↑ propionate in P1 ↑ total fatty acids, butyrate, and propionate in P2
Chi *et al*., 2021^75^ China OBS	GA: <37 weeks BW: <2,500 g Frequency: Daily for 12 weeks Dose: Not specified	PBX: *B. lactis* (*n* = 59) C: (*n* = 79)	α: ↑ β: Δ	↑ *Bifidobacterium* and *Lactobacillus* and ↓ *Enterococcus*, *Klebsiella*, and *Streptococcus*	Not assessed
Fleming *et al*., 2020^76^ United Kingdom RCT (MC, DB, PCT)	GA: <31 weeks Frequency: Daily until 36 weeks PMA Dose: 8.3–8.8×10^10^	PBX: *B. breve* BBG-001 (*n* = 13) C: (*n* = 16)	α: Not assessed β: Not assessed	↑ total bacteria, *Bifidobacterium* and *B. breve* BBG-001	Acetic, lactic and succinic acid ns
Hays *et al*., 2016^77^ France RCT (MC, DB, PCT)	GA: 25–31 weeks BW: 700–1,600 g Frequency: Daily for 4 (≤28 weeks GA) or 6 weeks (≥29 weeks) Dose: 1 × 10^9^	P1: *B. lactis* (*n* = 39) P2: *B. longum* (*n* = 40) P3: *B. lactis and B. longum* (*n* = 37) C: (*n* = 41)	α: ns β: Not assessed	↑ *Bifidobacterium* in PBX, particularly in P1 and P3	Fecal calprotectin ns
Millar *et al*., 2017^78^ United Kingdom RCT (MC, DB, PCT)	GA: <31 weeks Frequency: Daily until 36 weeks PMA Dose: 8.3–8.8×10^10^	PBX: *B. breve* BBG-001 (*n* = 48) C: (*n* = 40)	α: ns β: Not assessed	No differences	Not assessed
Underwood *et al*., 2013^79^ USA RCT	GA: <33 weeks BW: <1,500 g Frequency: P1 or P2 2× daily for 5 weeks with dose increases weekly Dose: 5.7 × 10^7−^4.2×10^9^ Frequency: P3 or P4 2× daily for 2 weeks each with 1 week washout in-between Dose: 4 × 10^9^	P1: *B. infantis* (*n* = 6) P2: *B. lactis* (*n* = 6) P3: *B.* *infantis* then *B. lactis* (*n* = 4) P4: *B. lactis* then *B. infantis* (*n* = 3)	α: ns β: ns α: ns β: ns	No differences between P1 and P2 ↑ Bifidobacteria and ↓ Gammaproteobacteria during *B. infantis* supplementation in P3 and P4	Not assessed
Lactobacillaceae					
Marti *et al*., 2021^80^ Sweden RCT (MC, DB, PCT)	GA: 23 to <28 weeks BW: <1,000 g Frequency: Daily until 36 weeks PMA Dose: 1.25 × 10^8^	PBX: *L. reuteri* DSM 17938 (*n* = 68) C: (*n* = 66)	α: ↑ β: Δ	↑ *Lactobacillus* and ↓ *Staphylococcus* and Enterobacteriaceae	Not assessed
Multi-Genera Bacterial Formulations					
Abdulkadir *et al*., 2016^40^ United Kingdom OBS	GA: <31 weeks Frequency: 2× daily until 34 weeks PMA Dose: 2 × 10^9^	PBX: *L. acidophilus* NCDO 1748 and *B. bifidum* NCDO 2203 (Infloran) (*n* = 7) C: (*n* = 3)	α: ↓ β: Δ	↑ *Bifidobacterium* and *Lactobacillus* and ↓ *Clostridium* and *Streptococcus*	Altered metabolite profiles
Alcon-Giner *et al*., 2020^39^ United Kingdom OBS (MC)	GA: ≤34 weeks Frequency: 2× daily until 34 weeks PMA or discharge if BW < 1,500 g Dose: 2 × 10^9^	PBX: *L. acidophilus* NCDO 1748 and *B. bifidum* NCDO 2203 (Infloran) (*n* = 101) C: (*n* = 133)	α: ↓ β: Δ	↑ *Bifidobacterium* and *Lactobacillus* and ↓ *Klebsiella*, *Escherichia, Enterobacter, Cutibacterium* and *Clostridium*	↑ acetic acid and lactate and ↓ 2’FL, 3’-FL, arabinose and trehalose ↓ fecal pH
Alshaikh *et al.*, 2022^42^ Canada RCT	GA: <29 weeks BW: <1,000 g Frequency: Daily until 37–39 weeks PMA Dose: 4 × 10^9^	PBX: *B.* *breve* HA-129,*B.* *bifidum* HA-132,*B.* *infantis* HA-116, *B.* *longum* HA-135 and *L.* *rhamnosus* HA-111 (*n* = 31) C: (*n* = 31)	Bacteria α: ns β: Δ Fungi α: ↑ β: Δ	↑ *Bifidobacterium* and *Lactobacillus* and ↓ *Candida*	See Samara *et al*., 2022
Beck *et al*., 2022^36^ United Kingdom OBS	GA: <32 weeks Frequency: Daily until discharge Dose: 2 × 10^9^	P1: *L. acidophilus* NCDO 1748 and *B. bifidum* NCDO 2203 (Infloran) (*n* = 24) P2: *B. bifidum, B. longum* subsp. *infantis*, and *L. acidophilus* (Labinic) (*n* = 71) C: (*n* = 28)	α: ↑ in P2 vs. C β: Δ	↑ *L. acidophilus, B. bifidum, B. breve* and *E. faecium* and ↓ *Veillonella parvula* and *Propionibacterium acnes* in P1 ↑ *B. bifidum, B. longum* subsp. *infantis, B. animalis*, and *L. acidophilus* in P2	Δ metabolic capacity by group Δ enzyme commission profile in PBX vs. C ↑ serum LPC in PBX
Chang *et al*., 2022^81^ Taiwan OBS	GA: >24 to ≤28 weeks Frequency: Daily for one week Dose: 2 × 10^9^	PBX: *L. acidophilus* NCDO 1748 and *B. bifidum* NCDO 2203 (Infloran) (*n* = 70) C: (*n* = 50)	α: ↑ β: Δ	↑ *Lactobacillus, Bifidobacterium bifidum*, *Faecalibacterium prausnitzii* and *Enterococcus faecium* and ↓ *Klebsiella*, *Serratia*, and *Staphylococcus epidermidis*	Inferential assessment
Gong *et al*., 2020^82^ China RCT	GA: 28 to 37 weeks Frequency: 2× daily for one week Dose: 3 × 10^7^	PBX: *B. longum*, *L. acidophilus*, *Enterococcus faecalis* (*n* = 20) C: (*n* = 20)	α: ↓ β: Not assessed	↑ *Bifidobacterium*, *Lactobacillus* and *Enterococcus*	Inferential assessment
Hui *et al*., 2021^38^ Denmark OBS	GA: <30 weeks Frequency: Daily for 30 days Dose: 2 × 10^9^ LGG and 2 × 10^8^ CFU/day BB-12	PBX: *Lacticaseibacillus rhamnosus* GG (LGG) and *Bifidobacterium animalis* subsp. *lactis* BB-12 (*n* = 87) C: (*n* = 165)	α: ns β: Δ	↑ *B. animalis*, Lactobacillales and *L. rhamnosus* and ↓ *Klebsiella, Veillonella* and *Weissella*	Inferential assessment
Kurath-Koller *et al*., 2020^83^ Austria OBS (MC)	BW: <1,500 g Frequency: Daily for 2 weeks Dose: 1 × 10^9^ P1 or 4 × 10^9^ P2	P1: *L. rhamnosus* LCR 35 (*n* = 18) P2: *B. infantis* NCDO 2203 *and L. acidophilus* NCDO 1748 (*n* = 18) C: (*n* = 18)	α: ns β: Δ	↑ *Lactobacillus* in P1 ↑ *Bifidobacterium, Lactobacillus* and *Geobacillus* in P2	Not assessed
Larke *et al*., 2022^84^ USA OBS	GA: 23 to 32 weeks BW: <2,500 g Frequency: Daily until 34 weeks Dose: 1 × 10^8^ P1 or 8 × 10^9^ P2	P1: *Lactobacillus reuteri* DSM 17938 (*n* = 16) P2: *Bifidobacterium longum* subsp. *infantis* EVC001 (*n* = 29) C: (*n* = 5)	α: Not assessed β: Δ	↑ Lactobacillaceae in P1 ↑ Bifidobacteriaceae in P2	↑ 2’-FL, 3’-FL, LNFP I-III, LDFT, LNT, 3ʹ-SL and 6ʹ-SL in P1 ↑ total fecal acids, 1,2-propanediol, formate, acetate, pyruvate, and indole-3-lactate in P2 ↓ fecal calprotectin in P2
Moreno-Sanz *et al*., 2022^85^ Spain RCT (DB, PCT)	GA: 28 to <31 weeks Frequency: Daily until 36 weeks PMA or discharge Dose: 1 × 10^9^ CFU/day *L. salivarius* and 1 × 10^8^ CFU/day *B. longum*	PBX: *L. salivarius* subsp. *infantis* PS11603 and *B. longum* PS10402 (*n* = 14) C: (*n* = 13)	α: ns β: Δ	↑ *Bifidobacterium*, *Lactobacillus* and Actinomyces and ↓ *Enterococcus* and *Coprococcus* ↑ *B. longum* and *L. salivarius* and ↓ *Enterococcus faecalis*, *Staphylococcus devriesei* and *Cutibacterium acnes*	↓ G-CSF, IL-17, and T_H_17 response
Plummer *et al*., 2018^86^ Australia RCT (MC, DB, PC)	GA: <32 weeks BW: <1,500 g Frequency: Daily until discharge or 40 weeks PMA Dose: 1 × 10^9^	PBX: *B. longum* subsp. *infantis* BB–02, *Streptococcus thermophilus* TH–4 and *B. animalis* subsp. *lactis* BB-12 (*n* = 38) C: (*n* = 28)	α: ns β: Not assessed	↑ *Bifidobacterium* and ↓ *Enterococcus*	Not assessed
Qiao *et al*., 2017 China^87^ RCT (DB, PC)	GA: <35 Frequency: 2× daily for 2 weeks Dose: 1.5 × 10^7^	PBX: *B. longum*, *L. acidophilus*, *Enterococcus faecalis* (*n* = 30) C: (*n* = 30)	α: Not assessed β: Not assessed	↑ *Bifidobacterium* and *Lactobacillus*	Not assessed
Samara *et al*., 2022^37^ Canada RCT	GA: <29 weeks BW: <1,000 g Frequency: Daily until 37–39 weeks PMA Dose: 4 × 10^9^	PBX: *B.* *breve* HA-129,*B.* *bifidum* HA-132,*B.* *infantis* HA-116, *B.* *longum* HA-135 and *L.* *rhamnosus* HA-111 (*n* = 26) C: (*n* = 31)	See Alshaikh *et al*., 2022	See Alshaikh *et al*., 2022	Compositional differences in metabolome ↓ IL-4, IL-10, IL-12 (p70), IFN-γ, and fecal calprotectin and ↑ IL-22
Underwood *et al*., 2009^88^ USA RCT (PC)	GA: <35 weeks BW: 750–2,000 g Frequency: Twice daily for 28 days or until discharge Dose: 5 × 10^8^	P1: *L. rhamnosus* GG and inulin (*n* = 30) P2: *L.* *acidophilus*, *B. longum*, *B. bifidum*, and *B. infantis* (*n* = 31) C: (*n* = 29)	α: Not assessed β: Not assessed	↑ *Bifidobacterium* in P2	Acetate, butyrate, and propionate ns
Van Best *et al*., 2020^35^ Germany OBS	GA: <32 weeks Frequency: Daily until 36 weeks PMA Dose: 2 × 10^9^ P1 or 4 × 10^9^ P2	P1: L. acidophilus ATCC 4356 and *B. infantis* ATCC 15697 (Infloran) (*n* = 28) P2: *L.* *acidophilus* La-14 ATCC SD5212, *B. longum* Bl-05 SD5588, *L. casei* ATCC SD5213 and *B. lactis* (Darmflora Plus) (*n* = 21) C: (*n* = 21)	α: ns β: Δ	↑ *Bifidobacterium, B. longum, B. infantis, Lactobacillus* and *Enterococcus and* ↓ *Klebsiella* in P1 ↑ *Enterobacter*, *B. lactis* and *L. acidophilus* and ↓ *Escherichia and Klebsiella* in P2	Not assessed
Watkins *et al*., 2019^41^ Ireland OBS	GA: <32 weeks Frequency: Daily until 34 weeks PMA (*n* = 10) or weekly for 4 weeks (*n* = 8) or bi-weekly for 4 weeks with 2 doses/week (*n* = 8) Dose: 2 × 10^9^	PBX: *L. acidophilus* NCDO 1748 and *B. bifidum* NCDO 2203 (Infloran) (*n* = 26) *C*: (*n* = 12)	α: ↑ with daily PBX β: Δ with daily PBX	↑ Enterococcacae and Streptococcaceae in PBX ↑ *Bifidobacterium* and ↓ Proteobacteria and Nocardiaceae in daily vs. weekly and bi-weekly PBX ↓ Lachnospiraceae, *Clostridium sensu stricto* and *Klebsiella* in weekly and bi-weekly PBX vs. control ↑ Bifidobacteriaceae in weekly PBX vs. control ↑ *Rhodococcus* in bi-weekly vs. daily PBX	Not assessed
Westaway *et al*., 2021^89^ Australia OBS	GA: 25 to <32 weeks BW: <1,500 g Frequency: Daily until 34–36 weeks PMA Dose: 2 × 10^9^	PBX: *L. acidophilus* NCDO 1748 and *B. bifidum* NCDO 2203 (Infloran) (*n* = 85) No control group	α: ns β: Δ	↑ *Enterobacter, Lactobacillus, Clostridium sensu stricto 1* and *Veillonella*	Not assessed
Westaway *et al*., 2022^90^ Australia OBS	GA: <32 weeks BW: <1,500 g Frequency: Daily until 34–36 weeks PMA Dose: 2 × 10^9^	PBX: *L. acidophilus* NCDO 1748 and *B. bifidum* NCDO 2203 (Infloran) (*n* = 14) C: (*n* = 4)	α: ↓ β: Δ	↑ *Bifidobacterium*, *Clostridium_M sp001517625* and *Flavinofractor plauti* and ↓ *Alistipes finegoldii*	Not assessed
Westaway *et al*., 2022^91^ Australia OBS	GA: <32 weeks BW: <1,500 gFrequency: Daily until 34–36 weeks PMA Dose: 2 × 10^9^	PBX: *L. acidophilus* NCDO 1748 and *B. bifidum* NCDO 2203 (Infloran) (*n* = 63) C: (*n* = 31)	α: ↑ β: Δ	↑ *Bifidobacterium, Lactobacillus Enterobacter, Cronobacter, Klebsiella, Veillonella* and *Clostridium sensu stricto 1* and ↓ *Streptococcus*	Functional genetic groups ns
Yousuf *et al*., 2020^34^ Canada OBS	GA: <32 weeks Frequency: Daily until discharge or hospital transfer Dose: 4 × 10^9^	PBX: *B.* *breve* HA-129,*B.* *bifidum* HA-132,*B.* *infantis* HA-116, *B. longum* HA-135 and *L. rhamnosus* HA-111 (*n* = 8) C: (*n* = 14) Term: (*n* = 51)	α: ns β: Δ	↑ *Bifidobacterium*, particularly *B. longum*	Not assessed
*Saccharomyces* spp.					
Costalos *et al*., 2003^92^ Greece RCT (DB, PC)	GA: 28 to 32 weeks Frequency: Daily for 30 days Dose: 1 × 10^9^	PBX: *S. boulardii* (*n* = 51) C: (*n* = 36)	α: Not assessed β: Not assessed	↑ Bifidobacteria and staphylococci and ↓ *Escherichia coli* and enterococci	Stool steatocrit and blood D-xylose ns
Zeber-Lubecka *et al*., 2016^93^ Poland RCT (DB, PC)	GA: 25 to 33 weeks Frequency: Daily for 6 weeks Dose: 2 × 10^9^	PBX: *S. boulardii* (*n* = 18) C: (*n* = 21)	α: ns β: ns	No differences	Not assessed

^a^Gestational age (GA) expressed in weeks. Birth weight only included if assessed as part of study inclusion criteria. Dose expressed as colony forming units (CFU) per day.

^b^Probiotic strain designations are included when reported in original manuscript.

^c^Only direct assessments of functional changes were included. Inferential assessments based on amplicon sequencing data were omitted.

Randomized controlled trial, RCT; observational study, OBS; multi-center, MC; double-blinded, DB; placebo-controlled, PC; gestational age, GA; postmenstrual age, PMA; birth weight, BW; colony forming units, CFU; probiotics, PBX or P; control, C; not significant, ns; fucosyllactose, FL; lacto-N-fucopentaose, LNFP; lactodifucotetraose, LDFT; lacto-N-tetraose, LNT; sialyllactose, SL; lysophosphatidylcholine, LPC; interleukin, IL; interferon, IFN.

### *Bifidobacterium* spp.

*Bifidobacterium* spp. have emerged as a leading candidate in probiotic development for infants and adults alike due to the extensive host benefits derived through their involvement in nutrient metabolism and cross-feeding network generation, production of antimicrobial peptides, and competitive exclusion of pathogens, among other health-promoting functions.^94^ The inclusion of bifidobacteria in probiotic formulations for infants is of particular interest due to the unique ability of certain strains to metabolize complex carbohydrates found in breast milk that infants are unable to digest, known as human milk oligosaccharides (HMOs).^95^ The production and transfer of HMOs from mother to infant via breast milk is a compelling example of human-microbe co-evolution, whereby HMOs produced by the mother function as an essential nutrient source in the infant gut, selecting for the proliferation and establishment of bifidobacteria.^95^ In healthy, breastfed infants born at term, bifidobacteria perform ecosystem services that promote optimal development and integrity of the gut microbiome.^96^ In their absence or reduced abundance, the course of early ecosystem establishment may be altered.^5,6,97^ Thus, a strong rationale exists for the inclusion of *Bifidobacterium* strains in probiotic preparations for preterm infants, particularly considering bifidobacteria are often absent or exhibit delayed colonization at low abundances in this population.^5–7,80^

Randomized controlled trials and observational studies of probiotics containing only *Bifidobacterium* strains vary considerably in terms of study design, strains investigated, duration of supplementation, and GA of participants at birth (Table 1), making it difficult to generate conclusive recommendations based on study-to-study comparisons. However, a common finding across these studies is the increased absolute and relative abundance of *Bifidobacterium* spp. in probiotic-treated infants, regardless of the strains included in the probiotic formulation.^74,75,77,79,84^ These infants also exhibit reductions in the abundance of potentially pathogenic bacteria, particularly Gammaproteobacteria,^74,75,79^ suggesting beneficial effects of bifidobacteria supplementation on the preterm microbiome. The taxonomic shifts observed with probiotic supplementation do not typically translate into changes in alpha-diversity,^74,75,77,78^ but are reflected in altered beta-diversity, highlighting the substantial effect of probiotics on the overall composition of the gut microbiome.^74,75^ These favorable results support the use of bifidobacteria-based probiotics for facilitating microbiome improvements in preterm infants. However, most of these studies have not evaluated the microbiome after probiotic supplementation has ended, limiting our understanding of the persistence of these alterations.

At the strain level, several studies have reported differences in the fitness of *Bifidobacterium* spp. to successfully colonize the infant gut and induce microbiome changes.^35,74,77,79^ For example, one RCT found that after 3 weeks of supplementation, preterm infants who received *B. lactis* (1 × 10^9^ colony forming units (CFU)) alone or in combination with *B. longum* (1 × 10^9^ CFU) displayed greater *Bifidobacterium* abundance than those who received *B. longum* alone.^77^ Comparable results have been reported in probiotic studies examining *B. breve* BBG-01 (8.3 × 10^10^ CFU)^78^ or *M*-16 V (3 × 10^9^ CFU)^29^ supplementation, including less substantial changes in the microbiome and stool metabolome when using single- vs. multi-strain probiotic preparations.^74^ The reduced fitness demonstrated by *B. longum* and *B. breve* when provided as a single strain may reflect their limited enzymatic repertoire for HMO metabolism and more fastidious nutritional requirements relative to other species of bifidobacteria.^98,99^ This is further evidenced by studies showing reduced maintenance of *B. longum* and *B. breve* after probiotic supplementation has ended,^34,35,37^ while *B. infantis* often persists in the infant gut,^35^ likely due to its ability to degrade all HMO types.^99^ However, culture-based experiments have shown that co-colonization of multiple strains results in higher bifidobacteria abundance and the generation of more resilient, interconnected communities due to increased metabolic cross-feeding of HMO derivatives.^95,100,101^ This is particularly beneficial to species, such as *B. longum* and *B. breve*, which have limited HMO metabolism abilities.^95,100,101^ This suggests that probiotics containing multiple strains of bifidobacteria may be more effective at generating beneficial shifts in the preterm gut microbiome, with evidence in support of this emerging in several clinical studies.^34,37,74,77^

Supplementation with probiotics containing bifidobacteria also results in functional shifts reflective of increased HMO metabolism and improved immune regulation. Metabolically, this includes elevated short-chain fatty acid (SCFA) concentrations,^74,76,84^ particularly an increase in acetate, and a reduction in fecal HMOs.^84^ Acetate is a primary by-product of HMO metabolism,^102^ with its production leading to reductions in intestinal pH and enhancements to gut epithelial defense mechanisms against pathogens.^103^ Thus, these metabolic shifts are congruent with the commonly reported reduction in pathobionts in infants supplemented with bifidobacteria.^74,75,79^ In parallel, supplementation with *B. infantis* EVC001 (8 × 10^9^ CFU) has been associated with lower levels of the inflammatory marker, fecal calprotectin, relative to other probiotic genera, suggesting bifidobacteria may promote a less pro-inflammatory state.^84^ However, this outcome was not observed across *Bifidobacterium* species, with *B. longum* (1 × 10^9^ CFU) and/or *B. lactis* (1 × 10^9^ CFU) supplementation having no significant effect on fecal calprotectin levels, despite using a comparable supplementation duration.^77^ Further research examining the metabolic and immune alterations arising due to the differential enzymatic capacities of various bifidobacteria strains in the preterm gut is needed. This will be crucial to informing the development of probiotic formulations optimized for the greatest benefit to the preterm population.

### Lactobacillaceae

Commercial interest in the development of probiotics containing lactobacilli is widespread in the nutraceutical industry, in part due to the long-standing role of these microbes in the production of fermented foods.^104^ Unlike bifidobacteria, lactobacilli are typically transient colonizers in the infant gut, often present immediately following birth in vaginally delivered infants due to vertical transmission from the vaginal canal.^49,105^ Although they do not serve as dominant members of the infant or adult gut microbiome, lactobacilli are often incorporated into probiotic supplements due to their ability to competitively exclude pathogens, antitoxic effects, immunomodulatory activities, and ability to metabolize HMOs, albeit to a lesser degree than bifidobacteria.^99,106,107^

In preterm infants, only one longitudinal RCT has evaluated microbiome outcomes resulting from supplementation with probiotics exclusively containing Lactobacillaceae (Table 1).^80^ This multi-site RCT provided extremely low birthweight infants (<1,000 g) born at <28 weeks GA with daily *Limosilactobacillus* (formerly *Lactobacillus*^108^) *reuteri* DSM 17938 (1.25 × 10^8^ CFU) from birth to 36 weeks PMA. In the first 4 weeks of life, probiotic-treated infants exhibited increased alpha-diversity, beta-diversity dissimilarity, and Lactobacillaceae abundance, alongside reduced abundance of Enterobacteriaceae and *Staphylococcus* spp. in the first week of life. The relative and absolute abundance of Lactobacillaceae gradually decreased over time, supporting the transient nature of lactobacilli colonization in the infant gut. At 2 years, no substantial differences in microbiome composition or diversity were observed between the probiotic and control groups.^80^ However, these transient microbiome shifts were positively associated with head growth at 36 weeks PMA and weight gain at 2 weeks, 4 weeks, and 36 weeks PMA, suggesting supplementation with *L. reuteri* DSM 17938 (1.25 × 10^8^ CFU) facilitates conditions conducive to infant growth.^80^

In small-scale observational studies, supplementation with Lactobacillaceae strains has resulted in comparable increases in the abundance of lactobacilli, yet the effects appear to be strain-specific.^80,83,84,88^ For example, *L. rhamnosus* LCR35 (1 × 10^9^ CFU) supplementation transiently increased lactobacilli abundance in the preterm microbiome, but this was not sustained at 2 weeks of age,^83^ and the effects of *L. rhamnosus* GG (5 × 10^8^ CFU) did not reach statistical significance.^88^ In contrast, more persistent increases in the abundance of lactobacilli have been observed with *L. reuteri* DSM 17938 (1–1.25 × 10^8^ CFU) supplementation.^80,84^ Despite having a reduced effect, treatment with *L. rhamnosus* LCR35 (1 × 10^9^ CFU) still resulted in compositional differences between probiotic and control groups, suggesting their effect on the microbiome is independent of successful colonization.^83^ These studies also reported that probiotic-associated changes in bacterial community composition were hospital-specific,^80,83^ highlighting the need to consider hospital-related effects on early colonization patterns, particularly by lactobacilli. It is possible that the effect of probiotic supplementation with strains of Lactobacillaceae may be more susceptible to differences in clinical environments and standard care practices between hospitals, including labor and delivery protocols, antibiotic use, and nutritional recommendations, among other factors.

Functionally, very little is known about the effects of treatment with Lactobacillaceae-based probiotics in preterm infants, with only one study examining microbiome, metabolomic, and immunologic measures.^84^ *L. reuteri* DSM 17938 (1 × 10^8^ CFU) supplementation was associated with increased levels of fecal HMOs and calprotectin relative to infants receiving *B. infantis* EVC001 (8 × 10^9^ CFU), indicative of superior HMO utilization and anti-inflammatory effects of bifidobacteria.^84^ These preliminary findings suggest that the beneficial effects of probiotic strains of Lactobacillaceae on the preterm gut microbiome and infant development are due to different underlying mechanisms than those described for bifidobacteria – possibly through competitive exclusion processes, rather than HMO metabolism and immune modulation. However, further research is needed to generate improved understandings of the role lactobacilli play in the preterm gut.

### Multi-genera bacterial formulations

Probiotics containing multiple genera, often of *Bifidobacterium* spp. and Lactobacillaceae, are the most frequently investigated formulations for infants born prematurely (Table 1). This is likely due to the potential for synergistic effects and broader health-promoting benefits achieved by including microbes with differing functional capacities. For the purpose of understanding distinct strain effects, this approach limits our ability to distinguish the strongest probiotic candidates for the amelioration of microbiome alterations in preterm infants, unless a factorial study design is used to permit estimating the individual and combined effects of different strains. However, the evaluation of multi-genera probiotic formulations has simultaneously been instrumental in revealing specific probiotic strains able to colonize the infant gut more successfully than others.^34,35,37,39,83^ For example, several studies have identified a decrease in Lactobacillaceae abundance as PMA increases and/or towards the end of probiotic supplementation, while *Bifidobacterium* spp. more often persist beyond this period.^34,35,37–41,80,83^ Given Lactobacillaceae are typically only abundant in the early postnatal weeks in term infants,^49,105^ prior to the dominance of obligate anaerobes (e.g., bifidobacteria), the reduction in their abundance may reflect typical successional patterns and not necessarily failed colonization. Therefore, it is important to consider both the confounding effects of multi-genera probiotics and the broader ecological context to generate clear understandings of how more complex probiotic formulations may contribute to ecological succession patterns in the preterm infant gut microbiome.

By far, the most-researched multi-genera probiotic formulation for use in preterm infants from a microbiome modification perspective is *L. acidophilus* NCDO 1748 (1 × 10^9^ CFU) and *B. bifidum* NCDO 2203 (1 × 10^9^ CFU) or Infloran, with several observational studies in Europe and Australia examining the effects of daily supplementation in preterm infants typically born at <32 weeks GA until 34–36 weeks PMA.^35,36,39–41,81,89–91^ In line with other probiotic studies, an increase in the abundance of the probiotic genera and a decrease in potentially pathogenic microbes have been reported in probiotic groups relative to controls.^39–41,81,90,91^ The effect of Infloran on alpha-diversity varied, including reports of increased,^81,91^ decreased,^39,40,90^ and unchanged^36,41^ alpha-diversity metrics between probiotic-treated and control infants. However, consistent alterations were observed in microbiome community composition (beta-diversity),^39–41,81,90,91^ similar to observations in single-genus probiotic studies.^22,74,80,84^ Notably, one small study examined the effect of daily (7 doses/week), weekly (1 dose/week), and bi-weekly (2 doses/week) supplementation with Infloran (2 x 10^9^ CFU) from birth to 34 weeks PMA to determine the minimum dosing interval required to effectively modify the preterm gut microbiome, revealing daily supplementation was necessary to induce significant and prolonged microbiome alterations.^41^ This included an increase in *Bifidobacterium* spp. abundance and changes in beta-diversity prior to 40 weeks PMA, indicative of accelerated microbiome maturation relative to infants who received probiotics weekly or bi-weekly.^41^ Distinct metabolic profiles were also observed between probiotic and control groups in several Infloran studies,^36,39,40^ including higher concentrations of acetic and lactic acid and lower fecal pH in probiotic-treated infants.^39^ Overall, these results are largely consistent with those of studies evaluating single-genus probiotics, showing beneficial taxonomic, compositional, and functional changes in probiotic-treated infants during the first weeks to months of life^35,36,39–41,81,89–91^ and that probiotic genera, particularly *Bifidobacterium* spp., may persist well-beyond the supplementation period.^36,39–41^

Other probiotic formulations containing a combination of one Lactobacillaceae and one *Bifidobacterium* spp. strain have also been evaluated in premature infants, revealing similar compositional findings to Infloran (*L. acidophilus* NCDO 1748 (1 × 10^9^ CFU) and *B. bifidum* NCDO 2203 (1 × 10^9^ CFU)) studies.^38,85^ One observational study of 252 infants born at <30 weeks GA receiving *L. rhamnosus* GG (2 × 10^9^ CFU) and *B. animalis* subsp. *lactis* BB-12 (2 × 10^8^ CFU) for 30 days after birth reported stronger maintenance of *B. animalis* abundance at the end of the supplementation period, while the abundance of *L. rhamnosus* declined.^38^ Notably, network analysis in this cohort revealed that phylogeny was the main determinant of microbial co-occurrence, with few cross-phylum patterns emerging, including for the probiotic genera. Instead, isolated network architectures were exhibited by most microbes.^38^ This finding further supports the idea that including multiple strains of the same genera(e.g., *Bifidobacterium*) in probiotic formulations may exert stronger influences on microbial community dynamics by facilitating the generation of within-phylum cross-feeding networks, while also suggesting probiotic strains of bifidobacteria and lactobacilli act independently of one another in the preterm gut.

Another small study (*n* = 27) of infants born at 28–31 weeks GA supplemented with *L. salivarius* subsp. *infantis* PS11603 (1 × 10^9^ CFU) and *B. longum* PS10402 (1 × 10^8^ CFU) daily until 36 weeks PMA or discharge reported lower levels of certain pro-inflammatory factors after 1 week of supplementation, but not at the end of the probiotic treatment. This included reductions in granulocyte colony stimulating factor (G-CSF) in meconium and interleukin (IL)-17 in feces.^85^ This study also reported several other trends suggestive of improved immune function in infants receiving probiotics,^85^ but considering its small sample size, immunological assessments in larger cohorts are needed to support the use of these probiotics for immunological benefits in preterm infants.

Head-to-head studies comparing different probiotic interventions have revealed distinct microbial signatures reflective of the probiotic formulation used.^35,36,83,84,88^ Notably, one observational study examining two probiotic combinations of Lactobacillaceae and *Bifidobacterium* strains, Infloran (*L. acidophilus* NCDO 1748 (1 × 10^9^ CFU) and *B. bifidum* NCDO 2203 (1 × 10^9^ CFU)) and Labinic (*L. acidophilus* (6.7 × 10^8^ CFU), *B. bifidum* (6.7 × 10^8^ CFU), and *B. infantis* (6.7 × 10^8^ CFU)), in infants born at <32 weeks GA showed that each formulation induced distinct microbiome maturational patterns relative to untreated controls.^36^ Using community typing analysis, both probiotics resulted in an increasingly mature community dominated by either *B. breve* in infants receiving Infloran (*L. acidophilus* NCDO 1748 and *B. bifidum* NCDO 2203) or *L. acidophilus*, *B. bifidum*, *B. infantis*, and *B. animalis* with Labinic (*L. acidophilus*, *B. bifidum*, and *B. infantis*) supplementation. In contrast, infants who did not receive probiotics transitioned toward communities dominated by *Klebsiella* spp. over the same time period.^36^ These differences were further reflected at the functional level, as fecal supernatants from each mature community cluster elicited differential effects on gene expression in experimental organoid monolayers.^36^ This study and other head-to-head studies also reported findings consistent with the aforementioned single- or multi-genera probiotic studies, highlighting the greater potential of *Bifidobacterium* probiotic strains to colonize and increase in abundance relative to Lactobacillaceae.^83,84,88^

Longitudinal studies by our group^37,42^ and others^34^ have examined multi-strain probiotics with increased formulation complexity in extremely preterm infants. These studies evaluated FloraBABY, a probiotic containing *B. breve* HA-129 (1.2 × 10^9^ CFU), *B. bifidum* HA-132 (8 × 10^8^ CFU), *B. infantis* HA-116 (6 × 10^8^ CFU), *B. longum* HA-135 (6 × 10^8^ CFU), and *L. rhamnosus* HA-111 (1 × 10^9^ CFU) from birth until 5–6 months corrected GA. Consistent with previous studies, the abundance of *Bifidobacterium* spp. and lactobacilli increased in probiotic groups in both studies, with only *Bifidobacterium* persisting after the supplementation period ended.^34,37^ Compositionally, the microbiome of supplemented preterm infants approximated that of term infants earlier than controls, indicating probiotics promoted accelerated microbiome transition toward a more mature gut microbial ecosystem,^34,37^ demonstrating greater stability and interconnectivity among species.^37^ Our group also examined the metabolome and inflammatory landscape in feces, revealing probiotic supplementation accelerated metabolic transition towards a term infant profile and facilitated a less pro-inflammatory state relative to controls, including significant reductions in interferon (IFN)-γ, IL-4, IL-10, IL-12 (p70), and calprotectin and an increase in IL-22 in probiotic-treated infants.^37^ Interestingly, we also observed a strong anti-*Candida* effect in the probiotic group independent of the accelerated bacterial microbiome maturation patterns, suggesting that probiotics may provide protection against common nosocomial infections through their influence on fungi.^37^ In summary, these studies emphasized the ability of multi-strain probiotic formulations to promote compositional and functional microbiome maturation toward a term-like state and an anti-inflammatory intestinal landscape in preterm infants. Although limited by small sample sizes (*n* = 57^37,42^ and *n* = 22^34^), these studies provide important clinical evidence of the sustained beneficial effects of this probiotic formulation in extremely preterm infants.

Probiotic formulations containing strains of *Streptococcus thermophilus* and *Enterococcus faecalis* in combination with Lactobacillaceae and/or *Bifidobacterium* spp. have also been evaluated in preterm infants.^82,86,87^ While these species are considerably less researched compared to lactobacilli and bifidobacteria, interest in their probiotic potential has increased due to their bactericidal, antitoxic, and pathogen exclusion properties.^109,110^ A longitudinal RCT of supplementation with *B. infantis* BB-02 (3 × 10^8^ CFU), *B. lactis* BB-12 (3.5 × 10^8^ CFU), and *S. thermophilus* TH-4 (3.5 × 10^8^ CFU) for 8 weeks reported increased relative abundance of *Bifidobacterium* spp. and reduced *Enterococcus* spp. in probiotic-treated infants over the course of the supplementation period, but not afterward, while *Streptococcus* spp. abundance did not change with the intervention.^86^ Similarly, two short studies in infants receiving probiotics comprising *L. acidophilus* (1 × 10^7^ CFU), *B. longum* (1 × 10^7^ CFU), and *E. faecalis* (1 × 10^7^ CFU) demonstrated increased absolute abundance of lactobacilli and *Bifidobacterium* spp. after 1–2 weeks of supplementation,^82,87^ with elevated *Enterococcus* spp. abundance and reduced species richness observed in one study.^82^ While limited by their short duration, these studies do not support the inclusion of *Streptococcus* and *Enterococcus* probiotic strains in formulations for preterm infants. In addition, longitudinal surveys of microbiome maturational trajectories during early life have consistently shown that *Enterococcus* spp. and *Streptococcus* spp. are abundant colonizers in very early stages of microbiome establishment in preterm and term infants, respectively,^5,6^ suggesting that supplementation with these species may delay transition to a more mature microbiome.

### *Saccharomyces* spp.

*Saccharomyces* spp. have been employed as a probiotic in both pediatric and adult populations for the treatment of gastrointestinal disorders, particularly diarrhea.^111–114^ Current evidence suggests that probiotic strains of *Saccharomyces* may confer benefits to the host through their diverse immunomodulatory effects, ability to sequester pathogenic bacteria, prebiotic effects of fungal cell wall components on SCFA production, and mutualistic interactions with the bacterial microbiome, all while being able to survive antibiotic treatments.^115–118^ Several studies have investigated the prophylactic potential of *S. boulardii* in preterm infants, revealing it may confer similar benefits to probiotic preparations containing bifidobacteria and/or lactobacilli with respect to NEC and LOS prevention.^119^ However, it remains largely unknown if these benefits are mediated in part through changes in the gut microbiome, as only two studies have examined microbiome measures. One RCT performed on infants born at 28–32 weeks GA reported decreased *E. coli* and enterococcci and increased bifidobacteria and staphylococci in infants receiving *S. boulardii* (1 × 10^9^ CFU).^92^ However, a more recent RCT using amplicon sequencing reported no differences in bacterial alpha-diversity, beta-diversity, or taxonomic structure after 6 weeks of *S. boulardii* (2 × 10^9^ CFU) supplementation.^93^ Given *Saccharomyces* spp. are associated with the introduction of solid foods^120^ and are typically absent or present in low abundances in the early postnatal period,^57,60,61^ particularly in preterm infants,^7,36,37^ their application from an ecological perspective is less justified. While the reported benefits of probiotic strains of *Saccharomyces* on pathogen adhesion and immunomodulation in other clinical populations are promising,^115–118^ further work is needed to understand the mechanisms underlying the prophylactic effects of yeast-based probiotics and their influence on the bacterial and fungal gut microbiome in infants born prematurely before recommendations regarding *Saccharomyces* supplementation can be made.

### Summary of current findings

Overall, the effects of probiotic supplementation in infants born prematurely appear to extend beyond the prevention of acute clinical conditions, having broad health-promoting influences on the gut microbiome, metabolome, and immune system. Both *Bifidobacterium*- and Lactobacillaceae-containing probiotics can modify the composition of the preterm gut microbiome, promoting a microbiome more representative of term infants.^37,39^ *Bifidobacterium* strains appear to have a superior ability to shift gut microbiome composition, stably colonize the infant gut beyond the supplementation period, and confer broad health benefits to preterm infants. This may be due to the diverse enzymatic repertoire encoded in bifidobacterial strains for HMO metabolism, resulting in the production of metabolites that facilitate the generation of cross-feeding networks.

Based on our results^37^ and other studies,^34,36^ we proposed that certain bifidobacterial strains can act as ecosystem engineers, capable of transforming the composition and function of the preterm infant gut microbiome, and thus, influence its maturational trajectory. However, these results do not indicate that Lactobacillaceae supplementation is unable to confer benefits to preterm infants, as evidence shows otherwise.^80^ It is possible that, despite not being prominent members of the preterm microbiome,^5,6^ the benefits of lactobacilli supplementation may be facilitated through their antimicrobial and competitive exclusion activities, effectively limiting the growth of pathobionts. Furthermore, probiotic supplementation may result in immunologic changes, such as reductions in the expression of pro-inflammatory cytokines (e.g., IFN-γ, IL-4, IL-10, and IL-12) and fecal calprotectin.^37,84,85^ While varying results have been reported,^37,84,85^ they reiterate the greater propensity for probiotic-supplemented preterm infants to achieve an anti-inflammatory immune environment when microbiome-modifying therapeutics are applied soon after birth. Future research into the mechanisms by which probiotics may confer these benefits is needed in order to better understand the type, dose, and timing of probiotic supplementation required to optimally influence ecological dynamics and the broader physiological state of preterm infants.

## Mechanisms of probiotic-induced shifts in the gut microbiome and host physiology

**Figure 2.**
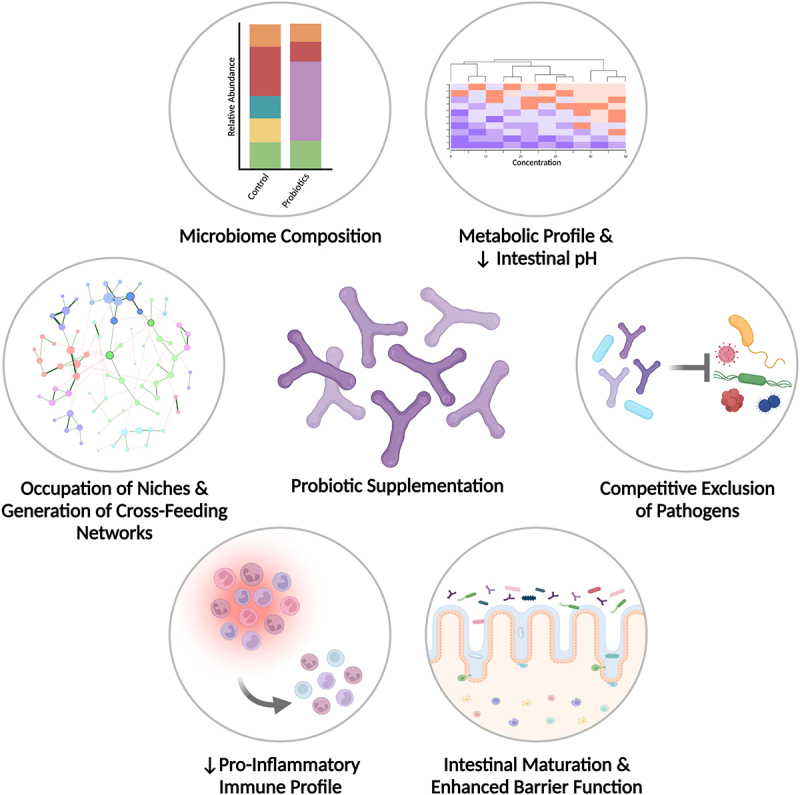
Potential mechanisms of probiotic-induced shifts in the gut microbiome and host physiology of preterm infants. Probiotic supplementation in preterm infants may influence gut microbiome maturation via several dynamically related mechanisms. First, probiotics can alter microbiome composition and initiate term-like maturation patterns, leading to the occupation of ecological niches and generation of cross-feeding networks amongst microbes. This facilitates alterations in the metabolic profile, and subsequently, a reduction in intestinal pH due to the greater production of acidic metabolites, such as acetic acid. The combination of microbial and metabolic shifts in the gut microbiome facilitates immune and intestinal maturation, enhancing gut barrier function and homeostatic defense mechanisms. This is further reflected by a reduction in pro-inflammatory immune markers, such as IFN-γ, IL-4, IL-12 and fecal calprotectin. Ultimately, the combination of these factors increase the resistance to pathogen colonization, while pathogens may also be competitively excluded from persisting in gut microbial ecosystems directly by probiotic strains. Thus, the provision of probiotics to infants born prematurely has the potential to significantly alter gut microbial ecology and broader host physiological measures to facilitate changes more representative of infants born at term.

The mechanisms by which probiotics may influence the gut microbiome and host physiology are diverse and dynamically related to one another. These include processes described above, such as altering the composition of the microbiome, competitive exclusion of pathogens by occupying vacant ecological niches, and shifting metabolic and immunologic profiles, alongside promoting maturation of the gastrointestinal tract and broader host physiological systems (Figure 2).^121^ Current understandings of the actions by which probiotics exert their effects have largely originated from mechanistic studies in animal models and culture-based experiments.^107,122,123^ Mechanisms have also been inferred from RCTs exploring probiotic supplementation for gastrointestinal disturbances in adults,^106,124^ but the transferability of these understandings to term and preterm infants is unclear. Knowledge generation efforts seeking to understand the mechanistic actions of probiotic supplementation in early life are important because modifications to an ecosystem in its primary phases of succession may have distinct and more pronounced influences than in a mature ecosystem.^125,126^ Despite this, continued learning from clinical studies in adults is warranted, as there are likely several shared mechanisms of probiotic action across age groups and clinical sub-populations.

A key mechanism by which probiotic supplementation influences the preterm microbiome and broader host physiology is through its impact on microbiome composition. Preterm infants are a unique clinical population displaying microbiome alterations representative of true dysbiosis, including the prolonged presence of skin- and hospital-associated aerobic bacteria that are not autochthonous members of the gut microbiome, such as *Staphylococcus* spp., and early dominance of potential pathobionts, such as *Klebsiella* spp., *Escherichia* spp., and *Enterococcus* spp..^5,6,52^ Unlike in other gastrointestinal disturbances, such as inflammatory bowel disease, where it remains disputed whether microbiome alterations are a cause or a consequence of disease,^127,128^ microbiome alterations in preterm infants appear to precede and contribute to the onset of acute clinical conditions such as NEC and LOS.^10–13,15–17,35^ Based on our recent finding that probiotic strains of *Bifidobacterium* and their associated metabolic by-products are stronger determinants of microbiome maturation than clinical or host factors,^37^ we propose that preterm infant gut microbiome dysbiosis is due to a fundamental inability to acquire these microbial strains in sufficient numbers to initiate term-like successional patterns. Therefore, early exposure to prospective colonizers through the provision of appropriate probiotic strains may be sufficient to induce shifts in microbiome composition away from pathobiont dominance and toward a more mature and stable microbiome comparable to term infants.

Probiotic-induced compositional alterations in the preterm microbiome subsequently elicit a cascade of health-promoting effects at the ecological, metabolic, and immune levels (Figure 2). From an ecological perspective, incorporation of probiotic species into resident gut microbial communities results in the occupation of vacant ecological niches and may contribute to the competitive exclusion of pathogens through the secretion of antimicrobial substances or inhibition of pathogen adhesion to the gut mucosa.^129,130^ For example, *in vitro* work has shown *that L. rhamnosus*, *B. breve*, and other probiotic strains can effectively inhibit and displace pathogens from adhering to the intestinal mucosa in a pathogen-specific manner, highlighting the importance of thoughtfully developed probiotic formulations considering common pathogens in the target clinical population.^107^

In parallel, probiotic strains may shift the metabolic state of the intestinal milieu and microenvironments, engineering new niches that may be colonized by successive members observed in the term microbiome. This facilitates the generation of cross-feeding networks that lead to greater interspecies connectivity and, therefore, stronger potential for robust competitive exclusion of pathogens.^95,100,101^ For example, the metabolism of HMOs by bifidobacteria results in the production of intermediary by-products, such as acetic and lactic acid, which may be further metabolized by butyrate-producing bacteria to generate cooperative networks of co-occurring microbes.^131,132^ Simultaneously, these processes effectively lower the intestinal pH to create an increasingly inhospitable environment toward pathogens.^130^ The production of SCFA, such as butyrate, may also enhance the structural maturation and integrity of the gastrointestinal tract given butyrate serves as an essential energy source for colonocytes and promotes the expression of tight junction proteins in intestinal epithelial cells, strengthening gut barrier function.^133,134^

Finally, the production of SCFAs and other organic acids, particularly by HMO-metabolizing bifidobacteria, may also improve homeostatic defense responses by immune cells and silence aberrant inflammatory processes,^135–137^ shifting the immunologic profile away from the pro-inflammatory state commonly observed in preterm infants.^72^ For example, in neonatal rats, bifidobacteria abundance has been associated with T cell development in the thymus and dendritic cell maturation in Peyer’s patches.^137^ Together, this cascade of cooperative mechanisms promotes accelerated maturation of both the microbiome and gastrointestinal environment, as well as broader host physiological measures, enhancing the resilience of the gut microbiome to perturbations. Continued investigations to generate strain-specific understandings regarding the mechanisms of action of probiotic bifidobacteria and lactobacilli will be important to increase our confidence in defining optimal probiotic formulations for use in preterm infants.

## Clinical considerations for probiotic supplementation in preterm infants

Current practices surrounding probiotic use in NICUs have engaged pediatric societies and researchers in complex, yet important, debates. There is no clinical consensus on who should be receiving probiotics, what probiotic formulations should be approved, when they should be introduced, and whether preterm infants, particularly those born extremely preterm (<28 weeks GA), should be receiving probiotics in the first place.^27–31,73,138^ Concerns regarding probiotic contamination and detrimental side effects, such as systemic infections, excessive immune activation, transfer of antibiotic resistance genes, and harmful metabolic activities, garnered substantial attention early on.^139–143^ However, accumulating evidence on the beneficial outcomes of including probiotic supplementation as part of standard care in many NICUs has alleviated these concerns, to a degree.^20–25^ Still, a cautionary approach to probiotic supplementation in preterm infants is warranted, particularly given their complex and highly individualized clinical course and the severity of potential outcomes if adverse side effects occur. Undeniably, the literature on probiotic supplementation in preterm infants has glaring deficits compared to that of other routinely used pharmaceuticals. Rigorously designed RCTs evaluating the timing of initiation, frequency, duration, mode of supplementation, and differences in outcomes among probiotic formulations are largely non-existent. Comprehensive evaluation of microbiome, metabolic, and immune parameters have also lagged, with most probiotic studies focusing on clinical outcomes, rather than the mechanisms by which they are achieved.^20–25^ Evaluations of changes in the composition and function of the gut microbiome, metabolome, and immune landscape (e.g., pro-inflammatory cytokines, calprotectin) with probiotic supplementation are needed, alongside translational studies in model systems (i.e., premature enteroids) to reveal the mechanistic actions of probiotic strains in the preterm gut. Without these understandings, probiotic supplementation in preterm infants will continue to involve an element of chance worthy of debate.

The clinical application of a nutraceutical or pharmaceutical product requires a high level of understanding of the parameters surrounding its use. Currently, RCTs examining variations of these parameters (timing of initiation, frequency, and duration) for probiotic use in preterm infants do not exist, yet observational studies suggest initiation of daily supplementation and higher probiotics doses (1 × 10^9^ to 2×10^9^ CFU/dose) soon after birth result in superior persistence of probiotic strains and beneficial shifts in gut microbiome composition.^41,79,84^ While the promotion of early initiation raises caution due to the risk of severe complications such as sepsis,^144,145^ providing autochthonous, non-pathogenic probiotic strains soon after birth enables immediate initiation of microbiome maturation patterns away from those that precede the onset of NEC,^10–13,15–17,35^ suggesting the benefits may outweigh the risks.

Our understanding of the duration of probiotic supplementation required to achieve the best clinical outcomes is also limited. For instance, most studies have used a daily supplementation approach for approximately 4–8 weeks (until 34–36 weeks PMA or hospital discharge; Table 1), which may not be necessary to achieve the desired outcomes in microbiome, metabolic, and immune parameters. However, studies on short-term probiotic supplementation demonstrating early compositional shifts in the microbiome lack longitudinal follow-up to determine if these alterations have long-term benefits.^38,81–83,87^ Furthermore, our understanding of strain-specific effects is lacking, with only a limited number of head-to-head studies examining differential outcomes resulting from supplementation with multiple probiotic products.^29,35,36,77,79,83,84,88^ As it stands, the optimization of probiotic use for preterm infants may not occur until longitudinal RCTs assessing these parameters are carried out, but current evidence suggests that reports of adverse events occurring with long-term probiotic supplementation in preterm infants are very uncommon.^20,25,146^

Besides administration parameters, nutritional considerations (i.e., human vs. formula milk, milk fortifiers, etc.) are warranted in identifying infants who may benefit most from probiotic supplementation. This was evidenced in a recent meta-analysis that found the preventative effects of probiotic supplementation on sepsis were specifically associated with human milk feeding,^147^ with similar findings existing for NEC.^23,148,149^ Human milk, including both mother’s own milk (MOM) and pasteurized donor milk, is a source of prebiotic HMOs that select for the proliferation of bifidobacterial probiotic strains with HMO enzymatic capacity.^95^ Given most infant formulas inherently lack these compounds,^150^ formula feeding may result in poor responses to probiotics due to the lack of adequate nutritional substrates required for bifidobacteria to thrive. Furthermore, it remains unknown if the demonstrated bifidogenic effect of HMO-supplemented formula use in term infants^151^ also occurs in preterm infants.

Clinical decisions regarding milk sources used in preterm infant feeding depend on several complex factors, including maternal production of breastmilk, breastfeeding intentions, clinical condition, and caloric intake requirements.^152,153^ Even if a mother is able to produce adequate amounts of breastmilk after a preterm delivery, its nutrient profile is often insufficient for preterm infants, resulting in the concomitant use of formula milk or milk fortifiers to ensure the infant’s caloric needs are met.^153–155^ Notably, two recent RCTs identified an association between human milk source (MOM vs. donor) and microbiome composition in very low birthweight infants (<1,500 g).^156,157^ The effect of milk type on the microbiome was dose-dependent,^157^ but inconsistent effects were reported for fortifiers (human vs. bovine).^156,157^ These results were recapitulated in an observational study of probiotic use in preterm infants that included formula fed infants, which found MOM and fortifiers significantly influenced the microbiome, although to a lesser degree than probiotics.^36^ Given these findings, the interactive effects of milk and fortifier types need to be considered in the context of probiotic supplementation and its influence on microbiome composition in preterm infants. This includes determining whether HMOs or other forms of prebiotics are required to achieve the desired benefits of probiotic supplementation on microbiome maturation and host physiological measures.

Currently, universal recommendations regarding probiotic formulations for worldwide use do not exist,^27,138^ in part reflecting the geographical influence on native strains present in the infant gut. Bifidobacteria appear to be important members of the microbiome in infants around the globe,^158–161^ and positive effects of probiotic supplementation have also been reported in low- and middle-income countries.^162,163^ However, the differential effects of probiotic strains need to be considered by pediatric societies and researchers alike when making recommendations in various geographical regions. This was highlighted by an RCT examining the use of a synbiotic containing *L. plantarum* ATCC 202195 (1 × 10^9^ CFU) and fructo-oligosaccharides (150 mg) in rural India, which found significant reductions in the incidence of sepsis and mortality in infants born at ≥35 weeks GA.^164^ The selection of *L. plantarum* ATCC 202195 was based on its greater potential to persistently colonize the neonatal gut relative to *L. rhamnosus GG* (1 × 10^9^ CFU) in previous India-based studies,^165,166^ suggesting that population-specific considerations are critical for the choice of effective probiotic strains.

Finally, probiotic supplementation has been strongly criticized for quality control issues, with the composition of probiotic formulations regularly reported to differ substantially from what is outlined on packaging.^167,168^ Since probiotics fall within the nutraceutical category, the associated development, manufacturing, and distribution processes lack the strict regulatory oversight of the pharmaceutical industry, which has raised highly warranted concerns among pediatric societies and researchers worldwide.^27^ Stringent quality control ascertaining the safety and efficacy of these products for use in preterm infants is necessary, particularly given their fragile physiological condition and higher likelihood of having indwelling medical supports that act as sites at risk of infection.^169,170^ While infrequent, reports of infections linked to contaminated probiotics exist,^171^ further emphasizing the need for the implementation of strict quality control measures to prevent these avoidable and potentially life-threatening clinical events. Recommendations regarding trusted probiotic formulations and suppliers, alongside regular quality control checks on probiotic composition and dose, are needed before probiotic supplementation becomes a routine part of clinical care.

## Conclusion and future directions

The adoption of probiotic supplementation into standard care practices for preterm infants has proven to be beneficial for the prevention of acute clinical conditions and the amelioration of microbiome alterations associated with prematurity. This includes the prevention of NEC, LOS, feeding intolerance, and all-cause mortality,^20–25^ alongside shifting the composition of the preterm microbiome toward one harboring fewer pathobionts and greater beneficial microbes. These microbiome modifications facilitate ecological, metabolic, and immune alterations within the host more reflective of infants born at term.^37,39^ Current evidence suggests probiotics containing bifidobacteria are superior to those containing lactobacilli or other probiotic strains for use in preterm infants, emphasizing the importance of ecological considerations in the development of probiotic formulations. Despite these advancements, our understanding of the complex dynamics that occur when supplementing a developing ecosystem with prospective colonizers and optimal guidelines surrounding probiotic use remain limited. To address this knowledge gap, probiotic intervention and observational studies evaluating microbiome, metabolic, and immunologic measures are needed. Specifically, evidence-based recommendations regarding probiotic use in preterm infants require thoughtfully designed RCTs evaluating the optimal timing of initiation, dosing, duration, mode of delivery, and composition of probiotic formulations.

Studies of probiotic supplementation in extremely (<28 weeks GA) and moderately (≥32 to <37 weeks GA) preterm infants are also limited, with most research to date focused on those born very prematurely (≥28 to <32 weeks GA). While extremely premature infants are at the highest risk of complications related to probiotic supplementation due to their vulnerable clinical condition, they may also reap the most benefits from early shifts in microbiome maturation patterns. Likewise, moderately and late preterm infants are often-overlooked sub-populations due to their less critical clinical condition, but may still benefit from probiotic use early in life given their reported microbiome differences relative to term infants.^5^ Ultimately, the absence of comprehensive microbiome and broader host physiological measures in probiotic studies to date represent a major limitation in our understanding of how the incorporation of probiotics into clinical care may benefit infants born prematurely. Only through longitudinal studies that capture these measures and longer-term health outcomes will we begin to understand if early-life microbiome modifications imparted through probiotic supplementation may impact the immune, metabolic, neurological, and cardiovascular health trajectories of children born preterm.
